# Necrotizing fasciitis as a rare complication of osteonecrosis of the jaw in a patient with multiple myeloma treated with lenalidomide: case report and review of the literature

**DOI:** 10.1186/2193-1801-3-123

**Published:** 2014-03-05

**Authors:** Patrizia Mondello, Vincenzo Pitini, Carmela Arrigo, Stefania Mondello, Michael Mian, Giuseppe Altavilla

**Affiliations:** Department of Medical Oncology, University of Messina, Messina, Italy; Department of Neurosciences, University of Messina, Messina, Italy; Department of Hematology & CTMO, Hospital of Bolzano, Bolzano, Italy; Department of Hematology & Oncology, Medical University of Innsbruck, Innsbruck, Austria; Via Lodi is. 47 b, 98124 Messina, Italy

**Keywords:** Necrotizing fasciitis, BRONJ, Bisphosphonate, Multiple myeloma, Lenalidomide

## Abstract

Bisphosphonates (BPs), potent inhibitors of osteoclast-mediated bone resorption, play a major role in the management of patients with multiple myeloma (MM). However, in the case of dental infections, they can lead to bisphosphonate related osteonecrosis of the jaw (BRONJ). This process can be worsened by concomitant antineoplastic therapy. Herein, we present a case of a life-threatening necrotizing fasciitis (NF) as a rare and severe complication of BRONJ after three cycles of lenalidomide and dexamethasone in an MM patient treated with corticosteroid therapy and Ibandronate for 5 years. The patient presented swelling on the right part of the neck, difficulty in swallowing and acute pain, so a magnetic resonance of the head and neck region was performed. It revealed the presence of an NF with a massive extension. Due to the large necrotic area and a rapid progression of the infection, the necrotic tissue had to be removed surgically. Furthermore, a specific antimicrobial treatment as well as 12 sessions of hyperbaric oxygen therapy were needed to cure the patient.

Herein, we highlight the potential serious adverse events associated with the use of bisphosphonates and antiangiogenetic drugs in patients with MM. Future studies are needed to evaluate the potential synergistic effects of BPs, corticosteroids and antiangiogenetic drugs.

## Introduction

Bisphosphonates (BPs) are important drugs in the treatment of neoplasia involving the bones. Particularly in multiple myeloma (MM) they are able to inhibit disease progression and even to prolong survival (Coleman et al. 2012). BPs are usually administered on a regular basis and often concomitantly with antineoplastic drugs such as thalidomide ore lenalidomide. However, in patients with infections involving the jaw, the administration of BPs can be complicated by a bisphosphonate related osteonecrosis of the jaw (BRONJ), a localized death of bone tissue with minor soft tissue involvement (Lee et al. 2014). Rarely, this severe complication can be further worsened by a necrotizing fasciitis (NF), a rapidly progressing infection characterized by extensive necrosis of subcutaneous tissue and fascia (Sultan et al. 2012; Tsitsilonis et al. 2013). If not promptly diagnosed and treated it can lead to a life-threatening condition.

## Case description

A 59-year-old woman was diagnosed with a Stage III MM IgA kappa. First line treatment consisted of three cycles of VAD (vincristine, doxorubicin and dexamethasone) followed by autologous stem cell transplant. Since the patient achieved only a partial remission, 5 months later she underwent 6 cycles of bortezomib monotherapy followed by radiation therapy of the whole dorsal column and pelvis. Having achieved a very good partial remission, monthly Ibandronate 6 mg and weekly 20 mg dexamethasone were delivered as maintenance treatment. Before and during BP treatment, the patient underwent a dental examination.

**Figure 1:**
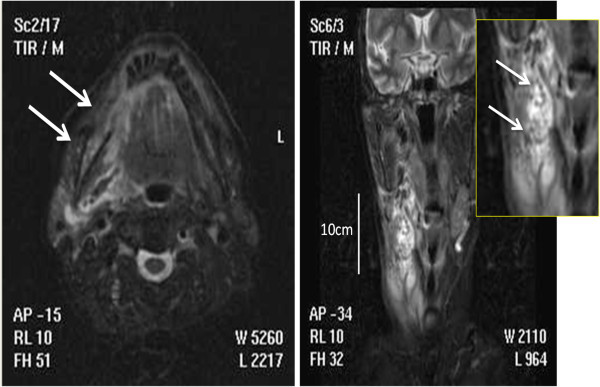
**Magnetic resonance imaging (MRI) study of head and neck. A**, Axial MRI scan showing a significantly increased signal of the bone marrow lesion (yellow arrow) when compared with left mandibular body (asterisk), with bone edema indicative of an inflammatory process taking place in the right mandibular body and gas tracking along the two pterygoid muscles (white arrows). **B**, A coronal T2-weighted image of the submandibular region showing a wide mass with very high signal intensity from the parapharyngeal space to the inferior cervical region with associated edema.

Two years later she presented with M-component in serum and urine, anemia and multiple lytic lesions. Therefore, second line treatment consisting of lenalidomide 25 mg/die, day 1–21 q28 and dexamethasone 25 mg/die p.o., Days 1–4, 8–11, 18–21) was initiated (Weber et al. 2007; Dimopoulos et al. 2007), while the BP was continued. After three cycles the patient presented swelling on the right part of the neck (from Robbinson level I to IV), difficulty in swallowing and acute pain. Intra-oral exploration revealed palato-pharyngeal paralysis on the right side and an ulcer of the right genua-mandibular mucosa with exposure of the surrounding alveolar bone. Within 24 to 48 hours from onset, local erythema, pain and edema quickly worsened. The skin appeared shiny and tense. The patient developed signs of systemic infection as well as atrial fibrillation. Routine laboratory tests revealed high levels of white blood cell count, creatine, C-reactive protein (CRP), and creatine kinase (CK). Magnetic resonance imaging (MRI) study of head and neck showed an increased signal of the right mandibular body, consistent with the diagnosis of right jaw osteonecrosis (Figure 1A), and a second high-intensity area characterized by gas formation spread for about 10 cm with involvement of the right parapharyngeal space and with extension to skin and muscle, suggestive of a gas-forming necrotising fasciitis of the neck (Figure 1B). Extension of the abscess was measured by computed tomography (CT) and ranged from the right side of the neck midline located at almost 1.5 cm from the cranial base to the upper edge of the right clavicle. The mediastinum was not involved. Surgical debridement with radical excision of necrotic tissues was necessary and histological examination revealed a lymphohistocytic infiltrate of the dermis, suppuration, necrosis of the superficial fascia, and edema in the fascial planes. Microbiological tissue cultures were positive for Streptococcus Mitis, a facultative anaerobe gram positive coccus that inhabits the human mouth. Combined antibiotic and antifungal therapy associated with nonsteroidal anti-inflammatories was initiated. Despite opioid-based pain therapy, total parenteral nutrition was necessary. In order to accelerate the healing process and because of the anaerobic infection, she underwent 12 sessions of hyperbaric oxygen therapy (OTI). Three months later pain was completely resolved and she was able to eat normally.

## Discussion

BRONJ is a severe complication of BPs therapy, not depending on the type of drug but on the duration of therapy since it accumulates in the bone tissue. Despite several cases of BRONJ having been reported (Lee et al. 2014), the pathophysiology of this complication is still unknown. However, a multifactorial genesis is strongly suggested. Recently, Vermeer et al. (2013) have shown that osteoclasts of the jaw and their precursors internalize a greater quantity of BPs compared to those of other bones. Since the inhibition of osteoclasts is dose dependent (Lam et al. 2007), bone remodeling is more reduced in the jaw than elsewhere in the body. Another effect of BPs is to promote a premature senescence of the oral keratinocytes (Kim et al. 2011a), impairing mucosal wound healing. Moreover BPs have antiangiogenic activity (Wood et al. 2002; Vincenzi et al. 2003) due to the reduction of circulating endothelial cells (Allegra et al. 2007) and by lowering VEGF serum levels (Santini et al. 2002). Beside BPs therapy, also corticosteroids, immunomodulatory drugs and chemotherapeutic agents have been implicated in the development of BRONJ (Saad et al. 2012) since most patients who have developed BRONJ have been treated with one or both. For example, thalidomide, an efficient antimyeloma agent,(Bamias and Dimopoulos 2005; Goranova-Marinova et al. 2009) has not only immunomodulary effects but also antiangiogenic activity contributing to impaired wound healing. Overall, the immunosuppressive effects of chemo- and radiotherapy, impaired bone remodeling due to corticoid therapy, and reduced vascularization due immunomodulatory drugs (IMiDs) are BRONJ favoring conditions (Goranova-Marinova et al. 2009).

In the present case, BRONJ was further complicated by NF, probably due to an over-infection of the necrotic tissue with successive extension to the surrounding structures. NF is a rare, rapidly progressing infection, characterized by extensive necrosis of subcutaneous tissue and fascia, usually accompanied by severe systemic symptoms (Sultan et al. 2012). It can occur de novo after inoculation of bacteria or as a complication of surgery or other traumas. Most frequent infectious agents are group A β-hemolytic streptococci, hemolytic staphylococcus, and Pseudomonas (Tsitsilonis et al. 2013). Although NF can occur in young and healthy patients, it usually afflicts elderly and/or immunocompromised patients. Early recognition of NF may be difficult because the initial clinical presentation is not specific and often resembles that of cellulitis (Chelsom and Halstensen 1994). An appropriate differential diagnosis between these two pathological conditions is critical for an effective clinical management. Indeed, while cellulitis, which is restricted to the subcutaneous tissue and can be cured in most cases with antibiotics alone, NF instead frequently requires an additional surgical intervention. The gold standard for the diagnosis of NF is a biopsy before surgery (Stamenkovic and Lew 1984). However, this procedure is invasive and can be associated with complications, therefore MR seems to be a valid alternative (Kim et al. 2011b). Both cellulitis and NF present a high signal intensity of subcutaneous tissue on T2-weighted images and a moderate to high contrast enhancement of the subcutaneous fat, but only in NF is there a deep fascia involvement identified in T2-weighted and contrast-enhanced T1-weighted images (Kim et al. 2011b). Moreover, as was the case with the herein presented patient, laboratory parameters such as CRP and CK could aid in early NF recognition since levels are higher in patients with NF than in those with cellulitis (Simonart et al. 2001). As in BRONJ, the combination of BPs anti-angiogenetic activity and lenalidomide, as well as the immunomodulatory effect of the latter could predispose the patient to NF. The vascular impaired and reduced viability of oral keratinocite slow down the reparative processes in the oral cavity and appear as predisposing factors in mucosal breakdown, facilitating bacteria infiltration. Moreover, patients with MM undergoing lenalidomide have an altered function of the immune system, promoting the extension of the infection. Indeed, Hsu et al. demonstrated that lenalidomide, in combination with dexamethasone (Len-Dex), leads to progressive reduction in the function of NK cells during the course of therapy (Hsu et al. 2011). As recently reported and similarly to our case, lenalidomide induced alterations of physiologic mechanisms can occur within a few days of the beginnig of treatment (Danbara et al. 2013).

The herein presented patient suffered from BRONJ followed by NF, both rare complications. Up to now their pathogenetic causes are not fully understood but a multifactorial genesis is strongly suggested. Available data implicates a major role of altered bone remodeling, reduced angiogenesis and therefore defective tissue repair mechanisms, altered microenviroment and immunodeficiency caused by antimyeloma therapy as well as the disease itself.

## Consent

Written informed consent was obtained from the patient for the publication of this report and any accompanying images.

## Authors’ information

**Patrizia Mondello, MD,** graduated with mark 110/110 cum laude in Medicine and Surgery at the University of Messina (Italy) in 2009 defending an experimental thesis entitled “Primary Lymphoma of the central nervous system” and is currently attending the fourth year of the post-graduate specialization in Oncology. She pursues different cancer specializations, focusing on the hematoncology field. She has received many awards and spent long periods in Germany and the USA, furthering her medical and anguage knowledge. She is currently spending a year at the Memorial Sloan Kettering Cancer Center in New York working in the laboratory of Dr. Anas Younes as research fellow and furthering her knowledge of translational research in the lymphomas field. She is also co-author of scientific papers published in national and international peer-review journals. Her main fields of research focus on pathophysiology in onco- and hematology fields and translational research.

**Vincenzo Pitini,** MD and Chief of “High dose chemotherapy and Stem cell transplantation department” at Universitary Hospital “G. Martino” in Messina. He graduated in Medicine and Surgery in 1977 with 110/110 cum laude. After graduation, he specialized in Renal, haematological diseases, and metabolic disorders in 1980. He specialized in Oncology in 1983. Since August 1980, he has been University Researcher at the Institute of Oncology at the University of Messina, and is still in service at the Department of Human Pathology. Since 1987 he has obtained the qualification of Aid. Since the academic year 1990/91 he has also given lesson cycles on the use of Molecular Biology in Oncology. Since 1995, he has developed the procedures for the collection and subsequent reinfusion of circulating stem cells in the high-dose antiblastic therapy, thereby contributing to the accreditation of the Division of Medical Oncology at the Italian Group for Bone Marrow Transplantation (GITMO) CIC 669. He also attended an updating course in Oncohematology at the University of Texas M.D. Anderson Cancer Center in Houston, Texas (USA). He is also author of scientific papers published on national and international peer-review journals focusing on hematology and oncology fields.

**Carmela Arrigo,** lab manager of Stem of “High dose chemotherapy and Stem cell transplantation department” at Universitary Hospital “G. Martino” in Messina. She graduated in Natural Science in 1975 and in 1986 in Medical Biology at the University of Messina. She specialized in “Medical Genetics” in 1993 at the University of Catania. She is also co-author of scientific papers published in national and international peer-review journals.

**Stefania Mondello**, MD, MPH, PhD, is a trained neurointensivist with an extensive experience in critical care, biomarker research and statistical analysis methods. She received her medical degree and completed her residency at the University of Messina. Afterwards she obtained her Master’s in Public Health at the University of Florida and then continued studies with a Ph.D. degree focusing on assessments of the clinical utility of brain damage biomarkers to assist in the management of severe traumatic brain injury patients. For the past 5 years she has been attending the Division of Critical Care Medicine at the University of Florida. Her clinical and PhD training qualified her to assume the responsibility of Director of Clinical Research at the biotech company, Banyan Biomarkers, Inc. Her research focuses on the use of biochemical markers to improved management, diagnosis and prognosis of patients including clinical validation and assessment of the relationships with clinical variables and physiologic monitoring. These research projects are being carried out in collaboration with NIH and DoD grants. Her intellectual contributions are documented in peer reviewed manuscripts, book chapters, abstracts, and presentations at international scientific meetings. She has been invited to serve on national and international grant review panels and has received a number of awards recognizing her contributions to the field. She was also recognized by the prestigious journal Nature.

**Michael Mian** graduated in Medicine at the University of Innsbruck in 2004. In 2005 he worked as resident at the University of Salzburg. In 2009 he specialized in hematology at the University of Verona. From 2009 to 2010 he was a research fellow at the the Institute of Oncology Research of the Istituto Oncologico della Svizzera Italiana for 1.5 years. During this ellowship he developed methods to interpret single nucleotid polimorphism array data together with clincal data. Since then, he has been working as a physician at the General Hospital of Bolzano. In collaboration with the International Extranodal Lymphoma Study group he provided new insights into the clinical behaviour of rare extranodal lymphoid malignancies.

**Giuseppe Altavilla** Full Professor and Director of Medical Oncology department at Universitary Hospital “G. Martino” in Messina and a current member of the national executive of the Italian Association of Medical Oncology (AIOM). He graduated in Medicine and Surgery in 1975. He specialized in Cardiovascular Diseases, Catholic University (Rome) in 1979 and in Oncology at the Catholic University of Rome in 1979. Since 1980 he has been Researcher at the Unit of Medical Oncology. He is Professor of the College of PhD in Neurooncology (cycles XXVIII to XXI). He is Professor of Medical Oncology at the School of Specialization in Oncology, Toxicology, Geriatrics, Radiotherapy, Physics, Nuclear Medicine, Internal Medicine, and Genetics. He is interested in the assessment of diagnostic and therapeutic protocols and the study of prognostic factors of various solid tumours and, recently in the pharmacogenomics of cancer. He is also author and co-author of numerous scientific papers published on national and international peer-review journals.

## Abbreviations

BPs: Bisphosphonates
BRONJ: Osteonecrosis of the jaws
NF: Necrotizing fasciitis
MM: Multiple myeloma
VAD: Vincristine, doxorubicin and dexamethasone
CK: Creatine kinase
CRP: C-reactive protein
MRI: Magnetic resonance imaging
CT: Computed tomography
OTI: Hyperbaric oxygen therapy
IMiDs: Immunomodulatory drugs
Len-Dex: Lenalidomide and dexamethasone.
